# The Role of Plant Transcription Factors in the Fight against Plant Viruses

**DOI:** 10.3390/ijms24098433

**Published:** 2023-05-08

**Authors:** Kotapati Kasi Viswanath, Song-Yi Kuo, Chin-Wei Tu, Yau-Heiu Hsu, Ying-Wen Huang, Chung-Chi Hu

**Affiliations:** 1 Graduate Institute of Biotechnology, National Chung Hsing University, Taichung 40227, Taiwan; viswanathkotapati@gmail.com (K.K.V.); unchatbleu124@gmail.com (S.-Y.K.); yhhsu@dragon.nchu.edu.tw (Y.-H.H.); 2 Ph.D. Program in Microbial Genomics, National Chung Hsing University and Academia Sinica, Taichung 40227, Taiwan; a0422777815@gmail.com; 3 Advanced Plant Biotechnology Centre, National Chung Hsing University, Taichung 40227, Taiwan

**Keywords:** transcription factor (TF), viral infection, NAC, MYB, WRKY, bZIP, AP2/ERF

## Abstract

Plants are vulnerable to the challenges of unstable environments and pathogen infections due to their immobility. Among various stress conditions, viral infection is a major threat that causes significant crop loss. In response to viral infection, plants undergo complex molecular and physiological changes, which trigger defense and morphogenic pathways. Transcription factors (TFs), and their interactions with cofactors and cis-regulatory genomic elements, are essential for plant defense mechanisms. The transcriptional regulation by TFs is crucial in establishing plant defense and associated activities during viral infections. Therefore, identifying and characterizing the critical genes involved in the responses of plants against virus stress is essential for the development of transgenic plants that exhibit enhanced tolerance or resistance. This article reviews the current understanding of the transcriptional control of plant defenses, with a special focus on NAC, MYB, WRKY, bZIP, and AP2/ERF TFs. The review provides an update on the latest advances in understanding how plant TFs regulate defense genes expression during viral infection.

## 1. Introduction

Crop growth and productivity are negatively impacted by a variety of abiotic and biotic stresses in the environment, including high temperatures, low-temperature, salinity, drought, pathogen infection, insect attack, and weeds [1,2]. These environmental constraints cause significant yield losses in major crops globally, amounting to more than 50% [3]. Consequently, major crops grown in the future are expected to be more vulnerable to a broader range and number of abiotic and biotic stresses, as well as their combinations.

Biotic stress, caused by living organisms such as viruses, bacteria, fungi, arachnids, and weeds, directly deprives plants of nutrients, resulting in reduced plant vigor and, in severe cases, plant death [4]. However, plants have evolved complex responses to biotic stress to maintain growth, yield, and survival [5]. Among the biotic stresses, viral infection is one of the most damaging and can severely inhibit plant growth and development [6].

Viruses, which are submicroscopic infectious agents, can infect all living organisms, including plants. They use a variety of strategies to replicate and spread within the plant’s cellular environment [7,8]. Viral infection causes local and systemic damage [9], but plants have evolved numerous physiological, metabolic, and molecular strategies to reduce the effects of viral stress [10,11]. Understanding the molecular principles and mining the stress-responsive genes that direct plant responses against viral stress is one of the foundations of developing stress-resistant crop varieties. Transcription factors (TFs) control the regulation of various genes; the roles of various TFs in the regulation of stress-responsive genes during bacterial and fungal infections have been extensively studied [12,13]. However, compared to those against other pathogens, the regulatory role(s) of TFs during viral infection is less understood. This review aims to curate and analyze the roles of different plant TFs and their regulation in defense responses against viral invasion in plants, based on the results of recent molecular analyses.

## 2. Signaling Cascade under Virus Entry

**Figure 1:**
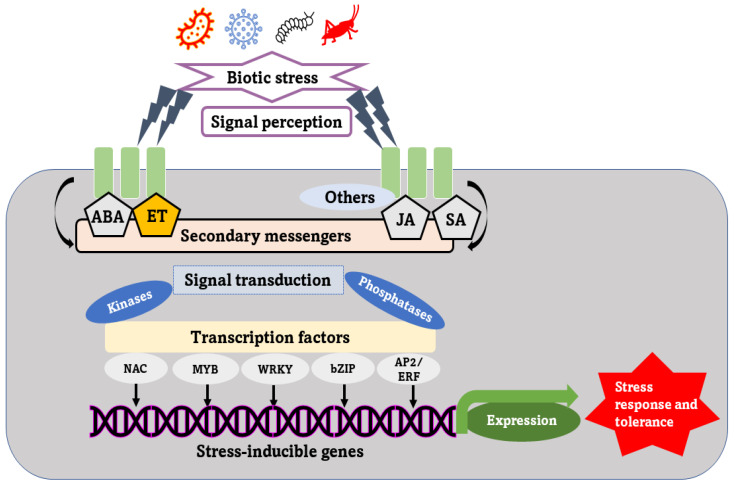
A schematic depiction of the general mechanism of biotic stress tolerance in plants. When plants are subjected to biotic stress, such as bacterial, viral, and insect infections, signal sensitivity is induced. It activates secondary mediators and regulates hormonal influences, causing transcription factors to be up- or down-regulated. Activated transcription factors will bind to specific promoter region cis-acting elements. The defense-related genes are activated by transcription factors with activation activity, leading to stress tolerance. The activity of defense-related genes is suppressed by transcription factors with repression activity, resulting in susceptibility to stress. ABA—abscisic acid; ET—ethylene; JA—jasmonic acid; SA—salicylic acid; NAC—no apical meristem, Arabidopsis-thaliana-activating factor, cup-shaped cotyledon; MYB—myeloblastosis-related transcription factor; WRKY—WRKY transcription factor; ERF—ethylene-responsive transcription factor; bZIP—basic leucine zipper transcription factor; bHLH—basic helix-loop-helix transcription factor.

Plants have evolved defense mechanisms that involve the recognition of pathogens and the production of a specific response in the plant’s cells. Cell wall receptors are responsible for this recognition, activating internal signaling components that initiate transcriptional and physiological changes within host cells (Figure 1). Plants and viruses may engage in several relationships, but if a pathogenic virus infects a plant, two innate immunity mechanisms may defeat the virus. Initially, pathogen-associated molecular patterns (PAMPs) are recognized by plant cell surface pattern recognition receptors (PRRs), which initiate PAMP-triggered immunity (PTI) that typically halts the infection of viruses before invasion [14,15]. The PRRs may trigger signaling cascades that launch transcriptional and physiological changes within host cells, eventually hindering pathogen growth and confirming PTI. RNA silencing, or RNA interference (RNAi), is a type of PTI against viruses [16]. To counteract this RNA-silencing mechanism, successful viruses deploy a range of effectors called RNA-silencing suppressors (RSSs) [17,18,19]. To counteract the RSSs effectors, the host plants have evolved factors that induce effector-triggered immunity (ETI) or resistance (R)-mediated defense mechanisms [17]. Many of these host factors possess DNA-binding domains, and have been shown to be transcription factors, as curated in the following sections. The ETI typically results in a hypersensitive response (HR), with localized cell death and defense gene expression, that inhibits virus growth and spread [20]. Genetic components that mediate HR are conserved across plant genera [21], and R-protein-mediated strategy works well against various pathogens, including viruses.

## 3. Transcription Factors Involved in the Virus Stress Responses

According to the Baltimore virus classification system [22], viruses may be categorized by their different types of genomes and mechanisms of gene expression. Most plant-infecting viruses are single-stranded (SS) RNA viruses (both positive-sense and negative-sense), and other types of viruses. The transcriptional control of defense-responsive genes is vital for plant stress response [23]. Transcription factors, which possess DNA-binding domains, play a significant role in controlling the transcription regulation and developmental processes, as well as responses to environmental cues in plants [24,25]. There are many different plant TF families, and six of them are predominantly involved in biotic and abiotic stress responses: NAC (no apical meristem (NAM), Arabidopsis transcription activation factor (ATAF), cup-shaped cotyledon (CUC)), MYB (myeloblastosis-related), WRKY (WRKY-domain containing proteins), bZIP (basic leucine zipper), AP2/ERF (Apetala2/ethylene-responsive factor), and zinc finger [12,24,26,27]. Studies have shown that the NAC [28], MYB [29], zinc finger [30], WRKY [31], AP2/ERF [32], bZIP [33], and bHLH [34] families of TFs are involved in the transcriptional activity of virus-responsive genes, such as R-gene [35], tobacco N-gene [36], genes linked to RNA silencing [37] and translation suppression [38]. The recent advances concerning the regulatory functions of NAC, MYB, WRKY, bZIP, and AP2/ERF TFs in various viral infections are summarized and discussed in the following sections.

### 3.1. NAC Transcription Factors Family

**Figure 2:**
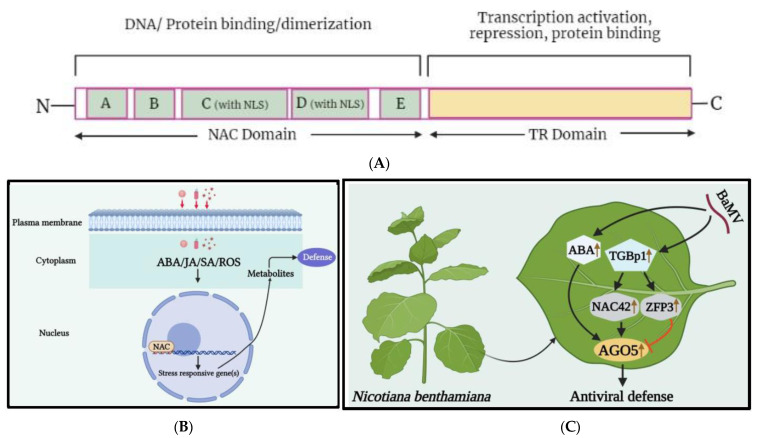
(**A**) A schematic representation of NAC transcription factors. A characteristic NAC protein has a highly conserved NAC domain at the N-terminal, which is further divided into five conserved sequence regions: A, B, C, D, and E. Regions A, C, and D are greatly conserved in diverse species, whereas regions B and E are comparatively variable. Regions C and D contain the predicted nuclear localization signal (NLS), which may be associated with the nuclear localization of transcription factors and identification of specific cis-acting elements in target gene promoter regions, while regions D and E are responsible for physical DNA binding [41,50]. The C-terminal region is more diverged and serves as a potential transcriptional regulatory (TR) domain, which has either activator or repressor function and may sometimes possess protein binding activity. (**B**) A basic depiction of NAC TFs mechanism under viral stress. When a virus infects a plant cell and releases viral particles, the cell responds with a hypersensitive response that disrupts its redox potential equilibrium. As a result, hormone-regulated pathways are triggered, leading to the activation of resistance genes. (**C**) The role of the transcription factors NAC and ZFP, as well as abscisic acid (ABA), in regulating NbAGO5 expression in response to *bamboo mosaic virus* (BaMV) infection. The expression of NbAGO5 is strongly induced after BaMV infection in *N. benthamiana*. NbNAC42 and NbZFP3 positively and negatively regulate the expression of NbAGO5, respectively. The plant hormone ABA, the production of which has been demonstrated to be stimulated by BaMV infection, also contributes to the stimulation of NbAGO5 promoter activity. Triple gene block protein 1 (TGBp1), which is encoded by BaMV, may regulate NbAGO5 expression and plant defense response by activating the NbNAC42 expression [51].

The NAC gene family is named after three TFs: NAM (no apical meristem), ATAF1-2 (*Arabidopsis thaliana* activating factor 1/2), and CUC2 (cup-shaped cotyledon 2), which share the same DNA-binding domain [39,40]. NAC genes are more specific to plants than to animals and have a highly conserved binding domain at the N-terminus, with a variable C-terminal domain that is crucial for transcriptional activity (Figure 2A) [41]. Advances in next-generation sequencing technology have led to the identification of numerous NAC TFs in various plant species. There are 19,997 NAC TFs from 150 species in the plant TF database, with the majority found in tomato, rice, Arabidopsis, and tobacco (http://planttfdb.gao-lab.org, accessed on 31 January 2022) [42,43]. Arabidopsis and rice NAC TFs are divided into 2 large groups and 18 subgroups [44]. Many NAC TFs bind to the stress-responsive DNA binding site, known as the NAC recognition sequence (NACRS), which contains the CGTG/A or CGT core consensus sequence [41]. Recent studies have shown that NAC TFs play a crucial role in regulating biotic stress responses in plants (Figure 2B) [45,46,47]. However, there are few reports on the functional role of NAC TFs during viral infection compared to plant growth, development, and abiotic stress [48].

In a genome-wide analysis of rice NAC TFs, researchers identified a total of 151 non-redundant NAC genes [43]. Within this gene family, a subgroup of NAC genes, known as SNAC genes, were found to be expressed in response to viral stress caused by *rice stripe virus* (RSV), *rice tungro spherical virus* (RTSV), *rice black-streaked dwarf fijivirus* (RBSDV), *rice grassy stunt tenuivirus* (RGSV), *rice ragged stunt oryzavirus* (RRSV), and *rice transitory yellowing virus* (RTYV). Microarray analysis revealed that nine SNAC genes were upregulated in response to RBSDV, RGSV, and RRSV, while the most upregulated SNAC genes were observed in response to RGSV, and the least expressed ones were observed in response to RTYV [49]. 

Although interactions between viral proteins and NAC TFs are rare, there have been some notable cases reported. For example, the *turnip crinkle virus* (TCV) capsid protein (CP) has been found to interact with the TCV-interacting protein (TIP), an Arabidopsis NAC transcriptional activator [52]. Loss of this interaction has been shown to reduce the ability of the TCV to induce HR and evade basal resistance, indicating that TIP is an essential component in the TCV resistance response pathway. Furthermore, the ability of TCV CP to bind TIP is associated with downregulation of the salicylic acid (SA)-mediated defense pathway, providing TCV with a replication advantage over TCV mutants, resulting in increased accumulation of wild type TCV early in the infection of TCV-susceptible Col-0 plants [53]. In wheat, geminivirus RepA-binding (GRAB) proteins, such as GRAB1 and GRAB2, have been identified as a novel member of the NAC domain family [54]. These proteins are capable of inhibiting geminivirus DNA replication in cultured cells, and the 37 amino acids from the C-terminal region of RepA protein encoded by *wheat dwarf virus* (WDV) are necessary for interaction with the N-terminal domain of GRAB proteins. These findings suggest that GRAB proteins may play a role in inhibiting the viral replication cycle. However, the underlying mechanism(s) of the inhibition remains to be investigated. It is possible that the interaction between host GRABs and viral RepA may trigger the transcriptional activation of other defense-related genes, or that the viral RepA protein might be sequestered by the host GRABs and viral replication might thus be interfered.

NAC083 is an essential component of geminivirus rolling circle replication in Arabidopsis, as it interacts with the replication initiator protein (Rep) of *mungbean yellow mosaic India virus* (MYMIV) [55]. However, the specific role of NAC083 in virus resistance has yet to be investigated. In tomato, the expression of six NAC TFs (SlNAC20, SlNAC24, SlNAC39, SlNAC47, SlNAC61, and SlNAC69) were found to be responsive to *tomato yellow leaf curl virus* (TYLCV) infection [28], with different expression profiles in virus-susceptible or -resistant cultivars. The silencing of SlNAC61 led to an increased accumulation of TYLCV DNAs, according to virus-induced gene silencing (VIGS) analysis, indicating that SlNAC61 serves a beneficial role in the defense against TYLCV infection. These NAC TFs interact with various defense response TFs, such as WRKY, MYB, and even NAC, by binding to their promoters, indicating a complex response mechanism during TYLCV infection.

The Arabidopsis NAC TF, ATAF2, interacts with the helicase domain of the *tobacco mosaic virus* (TMV) 126/183 kDa replicase protein [56]. ATAF2 is transcriptionally induced in response to TMV infection, and its overexpression increases the transcriptional activity of pathogenesis-related (PR) genes and reduces virus accumulation. ATAF2 functions in regulating host basal defense responses, as it shows increased transcript accumulation in inoculated tissues, but not in systemically infected tissues.

The *rice dwarf virus* Multiplication 1 (RIM1) gene in rice encodes a novel NAC-domain protein that belongs to the NTL subfamily and plays a role in the multiplication of *rice dwarf virus* (RDV) [57]. The *rim1-1* mutant, with an inserted retrotransposon Tos17 in the RIM1 gene intron, did not exhibit disease symptoms when infected with RDV and had a reduced accumulation of RDV CP. No similar effects were observed for RTYV and RSV infections. Therefore, it was suggested that RIM1 negatively regulates rice resistance to RDV by acting as a host factor that is necessary for the multiplication of the virus [57]. The *tomato leaf curl virus* (TLCV) infection upregulates the expression of *SlNAC1*, a member of the ATAF subfamily of NAC genes in tomato [58]. *SlNAC1* induction is mediated by geminivirus replication enhancer (REn) proteins, indicating its potential involvement in stress responses. Overexpression of SlNAC1 resulted in a significant increase in viral DNA accumulation, suggesting its crucial role in the process of REn-mediated enhancement of TLCV replication. In regal lily (*Lilium regale*), LrNAC35, a member of the ONAC022 subgroup in NAC family, was identified during *cucumber mosaic virus* (CMV) and TMV infection [59]. The *lily mottle virus* (LMoV) and *lily symptomless virus* (LSV) infections also triggered a significant increase in LrNAC35 transcripts in resistant and susceptible Lilium species. Overexpression of LrNAC35 in petunia enhanced resistance to CMV and TMV infections and promoted the accumulation of lignin in cell walls by regulating the Ph4CL lignin biosynthetic gene. NbNAC089, an endoplasmic reticulum (ER) membrane localized protein, with a transmembrane domain at the C-terminus, was discovered in *Nicotiana benthamiana* [60]. Upon TMV or CMV infection, the full-length NbNAC089 protein was cleaved and activated, and the protein may be translocated to the nucleus by releasing from the ER membrane. Knockdown of NbNAC089 increased the local and systemic movement of TMV-GFP, as well as TMV or CMV accumulation in *N. benthamiana*. NbNAC089 is suggested to be a negative regulator of unfolded protein response (UPR) and a positive regulator of programmed cell death (PCD), playing a crucial role in the defense of *N. benthamiana* to viral infection.

The *Capsicum annuum* NAC1 (CaNAC1) is a nuclear protein that contains the plant-specific NAC domain motif and has been identified as a TF involved in plant defense responses resulting in HR-cell death under *pepper mild mottle virus* (PMMV) invasion [61]. In watermelon infected by *cucumber green mottle mosaic virus* (CGMMV), 15 differentially expressed NAC TFs were observed, with 13 upregulated and 2 downregulated, possibly playing a role in the adaptation to CGMMV-induced stress in leaf tissues [59]. In maize, adenosylmethionine decarboxylase NAC TF was found to show a 300-fold upregulation under infection by *maize Iranian mosaic virus* (MIMV) and suspected to modulate maize defense responses to MIMV infection [62]. Previously, this TF was reported to interact with the nucleocapsid protein of *sonchus yellow net virus* (SYNV) in *N. benthamiana* [63]. Through RNA-seq analysis, upregulation of 7 differentially expressed genes (DEGs), including the NAC family transcription factors, was identified in passion fruit (*Passiflora edulis*) during the infection of CMV [64]. NAC1 TF was discovered through genetic regulation analysis using retrotransposons and suggested to play a critical role in hormonal regulation during *groundnut bud necrosis virus* (GBNV) and *tomato leaf curl New Delhi virus* (ToLCNDV) infection [65]. The citrus exocortis viroid (CEVd) infection in tomato resulted in overexpression of the ribosomal stress mediator, NAC082, which was associated with changes in ribosomal biogenesis and affected the development of 18S rRNA [66]. Transcriptomic data highlighted the significant upregulation of 88 NAC transcripts in the *sugarcane mosaic virus* (SCMV)-resistant (B-48) genotype of sugarcane compared to those of the susceptible (Badila) genotype [67]. Recently, we identified NbNAC42, an activator of argonaute 5 (NbAGO5) from *N. benthamiana* [51], which was significantly upregulated under *bamboo mosaic virus* (BaMV) infection, inducing the activity level of NbAGO5, and leading to antiviral defense (Figure 2C). Overall, the findings suggest that NAC TFs play a crucial role in regulating viral infection, multiplication, and accumulation by interacting with viral proteins or upregulating pathogen- or defense-related genes during viral infection. However, the underlying mechanism(s) for the seemingly contradictory roles of the various NAC TFs, in response to the infections plant viruses, remain to be thoroughly investigated.

### 3.2. MYB Transcription Factors Family

MYB TFs represent one of the largest gene families in plants and play a critical role in various biological processes, including plant growth and development, cell morphology, primary and secondary metabolic reactions, physiological activity metabolism, and responses to abiotic and biotic stresses [68,69,70]. The first MYB TF, v-Myb, was discovered in *avian myeloblastosis virus* (AMV) [71], and subsequent research has found MYB TFs in fungi, slime mold, animals, and plants [70]. The first plant MYB gene, maize *COLORED1* (C1), was found two decades ago, and its expression of MYB domain protein is necessary for anthocyanin synthesis in the maize aleurone [72]. The MYB TF is characterized by its highly conserved MYB (Myeloblastosis) DNA binding domain (DBD), which repeats at the N-terminal region and is evolutionarily conserved in nearly all eukaryotes [69,73].

**Figure 3:**
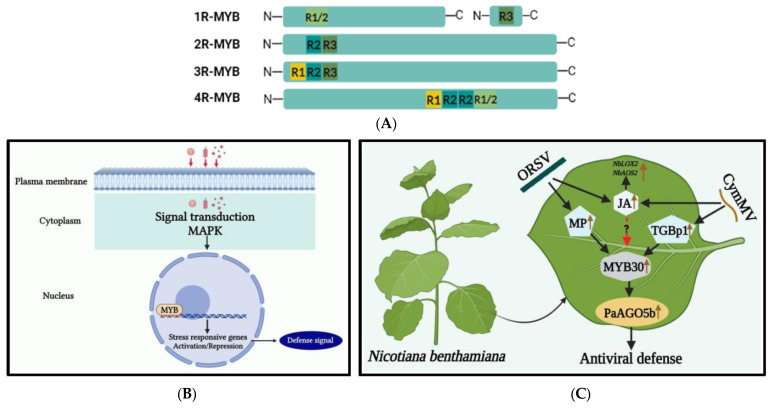
(**A**) Illustration of the classification and structure of myeloblastosis-related (MYB) TFs in plants. MYB TFs with one to four MYB domain repeats that are identified in plants. (**B**) Mechanism of MYB TFs under viral stress. During viral stress, a cascade of signaling mechanisms, leading to plant defense responses, is triggered as modulation of very long fatty acid chains takes place, mounting a HR response within the plant cell. MYB TF interacts with promoter elements of defense-related genes, which plays a crucial role in this response. (**C**) A model for enhancing antiviral defense mechanisms through the activation of the *Phalaenopsis aphrodite* subsp. *formosana* argonaute 5b promoter (pPaAGO5b). The *Cymbidium mosaic virus* (CymMV) and the *Odontoglossum ringspot virus* (ORSV) infectious clones and their triple gene block protein 1 (TGBp1), a CymMV-encoded protein, and movement protein (MP), an ORSV-encoded protein, may up-regulate plant defense-related NbMYB30. The NbMYB30 binds to the *p*PaAGO5b (*p*-indicates the promoter) and transcriptionally activates *PaAGO5b* expression to strengthen the antiviral defense mechanism. During viral infection, the phytohormone JA and related marker genes (NbLOX2 and NbAOS2) were upregulated. LOX2-lipoxygenase 2; AOS2-allene oxide synthase 2 [79].

The DBD of MYB consists of about 52 amino acid residues that fold into three α-helices, which are named R1, R2, and R3 due to their resemblance to the c-Myb protein. Based on their DBD structure, MYB TFs in plants are divided into four subfamilies: 1R-MYB, R2R3-MYB (2R-MYB), R1R2R3-MYB (3R-MYB), and 4R-MYB [74] (Figure 3A). The C-terminal region of MYB proteins is more variable than the N-terminus and is responsible for regulatory function. The R2R3-MYB subfamily, which contains two repeats, plays a crucial role in signaling various stresses, including biotic and abiotic ones [75,76]. Overexpression of some R2R3-MYB TFs activates plant PR genes, triggering systemic acquired resistance (SAR) against biotic stresses. This response is modulated by phytohormones, particularly jasmonic acid (JA) and salicylic acid (SA) [77,78]. Relatively little was known concerning the roles of MYB TFs involved in plant defense responses against virus invasion, as reviewed below. The molecular mechanism of MYB TFs at the time of viral infection is shown in Figure 3B. 

During TMV infection in tobacco (*N. tabacum*), the induction of NtMYB1 has been observed, leading to HR and development of SAR in the resistant tobacco cultivar due to the rise of endogenous SA [29]. However, NtMYB1 was not activated in the susceptible cultivar, which fails to accumulate SA. Exogenous SA treatment activated the expression of NtMYB1 in both resistant and susceptible tobacco cultivars, leading to the induction of PR genes several hours later. The suppression of NtMYB1 has been shown to compromise N-mediated resistance to TMV, confirming the role of NtMYB1 in R-gene-mediated resistance [80]. Tomato MYB28 TF (SlMYB28), an R2R3-MYB TF, was strongly induced by TYLCV infection in both susceptible and resistant cultivars [81]. Silencing SlMYB28 improved tomato plant resistance to TYLCV infection, suggesting that it acts as a negative regulator for viral infection control. In tomato plants infected with *tomato leaf curl virus* (ToLCV), lower levels of MYB33 TF have been observed, which is due to the two-fold higher accumulation of miR159a levels [82]. The MYB33 TF control gene expression of numerous components involved in floral, anther and leaf development. Upon *cauliflower mosaic virus* (CaMV) gene VI (P6) expression in Arabidopsis, upregulation of AtMYB96 was reported, and a subgroup of PR genes was also upregulated due to AtMYB96 overexpression [83,84]. In watermelon (*Citrullus lanatus*), 18 differentially expressed MYB TFs have been discovered during CGMMV invasion [85]. Among these, 15 and 3 MYB TFs were upregulated and downregulated in watermelon leaf tissues, respectively. Furthermore, after CGMMV infection, a watermelon MYB gene, Cla017179, was generally upregulated in leaves and fruit, implying that the MYB gene may play an important role in the response to CGMMV infection.

The *Antirrhinum majus* MYB-related TF Ros1 initiates the biosynthesis of colored anthocyanins and is used as a reporter system to track its expression [86]. The infiltration of *N. tabacum* plants with TEV clones resulted in a bright red pigmentation upon viral infection. This visual marker system facilitates quantitative and qualitative analysis of viral load through a simple extraction process. The Ros1-based reporter system has been successfully used to track the infection and movement of several plant viruses in different host plants, such as *turnip mosaic virus* (TuMV) and *potato virus* X (PVX) in Arabidopsis and *N. benthamiana*, respectively. TEV is a popular model system for studying positive-sense RNA viruses in plants, and changes in gene expression profiles have been observed upon TEV infection [87]. 

Overexpression of OsMYB4 in rice induced the expression of multiple genes in Arabidopsis and tomato involved in resistance to *tobacco necrosis virus* [88]. Functional analysis suggests that rice *OsMYB4* plays a critical role in the interplay of stress signaling pathways, as it activates many components, but its activity depends on the host’s genetic makeup [89]. In cucumber, six differentially expressed MYB TFs have been identified after infection with CGMMV [90]. The expression of these MYB TFs were upregulated early after infection compared to NAC and bZIP TFs, highlighting their importance under CGMMV infection. A genome-wide analysis of R2R3-MYB TFs in sugarcane (*Saccharum officinarum*) revealed the identification of a total of 202 R2R3-MYB genes (356 alleles) [91]. After SCMV infection, the expression of 10 R2R3-MYB genes increased, while one gene, MYB176, decreased, suggesting that these MYB genes are involved in response to SCMV infection [91]. 

The elevated expression of *Thinopyrum intermedium* MYB TF (TiMYB2R1) in wheat demonstrated its involvement in disease resistance by gradually increasing defense-correlated genes within the transgenic plant [92]. In Arabidopsis, the L10-Interacting MYB (LIMYB), a leucine-rich, receptor-like kinase (LRR-RLK), interacts with NIK1, causing translational suppression. Transcriptional repression of ribosomal protein genes, caused by overexpression of LIMYB, leads to protein restriction, reduced viral messenger RNA interaction with polysome fractions, and increased tolerance to begomovirus. Conversely, the repression of translation-related genes is released, increasing susceptibility to viral infection when LIMYB function is lost [38]. In *N. benthamiana*, the MYB4-like TF, coupled with the ethylene pathway, contributes to viral resistance. During TMV infection, NbMYB4L transcription is upregulated, and silencing the gene increases susceptibility to TMV replication [93]. The key TFs MYBPA1 and MYBA, which regulate the anthocyanin biosynthesis gene, are strongly repressed under *grapevine leaf-roll-associated virus*-3 (GLRaV-3) infection in Vitis vinifera [94].

Recently in our studies, the defense mechanism against CymMV and ORSV in *Phalaenopsis aphrodite* subsp. *formosana* was studied [79,95]. The PaAGO5b protein was found to play an important role in defense against orchid-plant-infecting viruses. Additionally, transcriptome analysis revealed that NbMYB30 and PaMYB30 were upregulated during CymMV and ORSV infections. The silencing of NbMYB30 resulted in increased virus accumulation in both *N. benthamiana* and *P. aphrodite* subsp. *formosana*. The upregulation of hormonally related TFs, such as NbMYB30, was suggested to control CymMV infection by increasing levels of JA and its marker genes, LOX2 and AOS2 (Figure 3C).

### 3.3. WRKY Transcription Factors Family

WRKY TFs play a crucial role as transcriptional regulators in plants, with multiple members identified in various species. For instance, 197 WRKY TFs have been discovered in soybean, 109 in rice, 74 in Arabidopsis, 83 in tomato, and 61 in cucumber [96,97,98,99]. These WRKY TFs play a prominent role in several physiological processes, including growth, metabolism, and the response to biotic and abiotic stresses [100,101].

**Figure 4:**
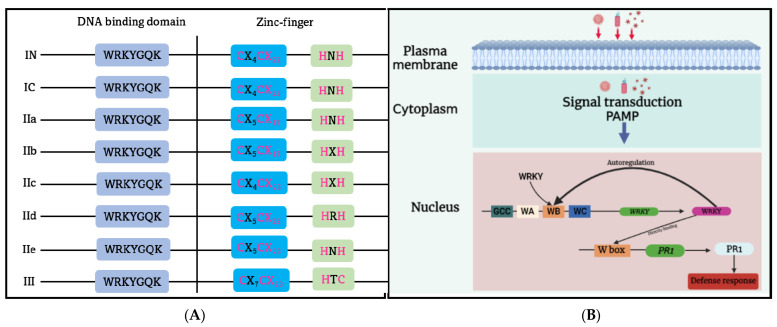
(**A**) The simplified illustration of the plant WRKY gene families. The WRKY gene family is classified into the I (I N and I C), II (IIa, IIb, IIc, IId, IIe), and III subfamilies. (**B**) A diagram that illustrates the participation of WRKY1 in pathogen-associated molecular pattern (PAMP)-induced regulation of the WRKY gene and its target gene through a negative feedback loop or direct binding to the W box of the pathogen-related gene 1 (PR1) gene. WA, WB, and WC represent a specific arrangement of the W box in the promoter of WRKY1, interacting with constitutively expressed WRKY proteins.

The WRKY domain (WD) is a highly conserved DNA binding domain found in WRKY protein families. It consists of a WRKY-like motif, WRKYGQK, at the N-terminus, followed by diverse downstream zinc finger motifs [102]. WRKY TFs can be divided into three major groups, based on structural diversity and phylogenetic analysis [103,104] (Figure 4A). Group I members have the Cys2-His2 zinc finger (C2H2 ZF) motif and typically have two WRKY domains located in the N- and C-termini, respectively. Those in Group II contain one WD and one C2H2 ZF motif, and can be further classified into subgroups IIa, IIb, IIc, IId, and IIe, based on the variation in ZF motif length and the polymorphism of key amino acid residues in WD [101]. Group III WRKY TF has a distinct C2HC ZF motif and a single WD, unlike the other WRKY groups. Generally, WD recognizes the W-box cis-element (TTGAC(C/T)) on the target DNA molecule. The WRKY protein’s C-terminal β-sheet structure can bind to the major groove of DNA, and the WRKYGQK motif’s Arg-415, Lys-416, Tyr-417, Gly-418, Gln-419, and Lys-420 residues can interact with the W-box via polar and hydrogen bonds with thymine methyl groups [105].

WRKY TFs have been shown to play an important role in responding to biotic stress and influencing plant defense mechanisms, as shown in Figure 4B. They can activate innate systems such as PTI and ETI or coordinate with other TFs to activate downstream defense genes. Studies suggest that WRKY TFs regulate phytohormone-mediated pathways that contribute to plant defense [106]. For instance, WRKY70 has been shown to regulate both SA- and JA-mediated defense signaling. Silencing WRKY70 activates JA-responsive/Coronatine-insensitive protein 1 (COI1)-dependent genes, but overexpression of WRKY70 reinforces resistance against pathogens and leads to the expression of SA-induced genes [107]. Additionally, the W-box sequence in the promoter region of NONEXPRESSOR OF PR1 (NPR1), a well-known regulator of the SA signaling pathway, is controlled by WRKY during activation of the plant defense response [108].

While most studies have concentrated on the role of WRKY in response to bacterial or fungal attack, an increasing amount of research indicates that WRKY can also react to viral infection via a conventional signaling pathway, making it another crucial determinant in the accumulation of viruses. A comparative transcriptome analysis of TYLCV-resistant and -susceptible tomato cultivars, Zheza-301 and Jinpeng-1, respectively, revealed that six group III WRKY TFs responded to TYLCV infection: WRKY41, WRKY42, WRKY53, WRKY54, WRKY80, and WRKY81 [109]. In infected Jinpeng-1 plants, all six WRKY TFs were upregulated compared to non-inoculated samples, whereas only WRKY42 and WRKY80 were upregulated in infected Zheza-301 cultivar. Silencing of either WRKY41 or WRKY54 in Zheza-301 led to a reduction in TYLCV DNA accumulation, and interaction network analysis showed that WRKY41 interacted with *isochorismate synthase* (ICS), which is required for the synthesis of SA, through the interaction with W-box in the ICS promoter. WRKY80 interacted with MAPK5, which can activate several TFs involved in resistance against pathogens. These findings suggest that these WRKY TFs are involved in regulating defense mechanisms against viral pathogens. During *South African cassava mosaic virus* (SACMV) infection, several WRKY TFs expressed differentially in cassava (*Manihot esculenta* Crantz) [110]. WRKY11 (MeWRKY11) was upregulated at 32 dpi, while MeWRKY81 was specifically down-regulated at 67 dpi in the cassava mosaic disease (CMD)-resistant landrace, TME3. On the other hand, MeWRKY27 and MeWRKY55 were both upregulated at every time point in the CMD-susceptible landrace, T200. Further gene ontology (GO) analyses on their homologs in Arabidopsis indicated that these MeWRKY TFs may regulate hormone signaling in plants, including JA, abscisic acid (ABA), ethylene (ET), and SA, which could determine the resistance of plants to biotic stress. *Mulberry mosaic dwarf-associated virus* (MMDaV)-encoded RepA was found to trigger HR through the upregulation of NbWRKY1 transcript [111]. Although no direct interaction was observed between RepA and NbWRKY1, the significant antiviral resistance conferred by NbWRKY1, and its nuclear localization, suggested that NbWRKY1 may enhance the resistance of plants by regulating the downstream network against MMDaV. Furthermore, RepA expression provides plant resistance to two begomoviruses in *N. benthamiana*, suggesting that responses of plant to RepA might be used to increase plant immunity to geminivirus attacks in crops [111].

The significance of WRKY TFs in the regulation of plant defense is well established. WRKYs are involved in both TIR (Toll-interleukin-like receptor) and non-TIR resistance genes, which encode proteins containing NBS-LRR domains [112]. In tobacco plants, WRKY TFs play important roles in the N-mediated resistance pathway against TMV. The silencing of NbWRKY1, NbWRKY2, or NbWRKY3 conferred the N-mediated restriction on viral systemic movement, and NbWRKY1 silencing resulted in smaller lesion sizes in inoculated leaves, demonstrating the importance of these NbWRKYs in the HR and SAR against TMV [80,113]. Another example of WRKY’s involvement in R gene-mediated defense against viruses is in the extreme resistance in soybean (Glycine max) conferred by the non-TIR R gene Rsv1 against the *Soybean mosaic virus* (SMV) [114]. The silencing of GmWRKY6 or GmWRKY30 resulted in the recovery of infection foci on the Rsv1 line, demonstrating the significance of WRKY in this defense mechanism. In *N. tabacum*, the WRKY TF TIZZ was identified as a TMV-induced, HR-responsive gene that contains a C2H2 ZF motif and a single WD. TIZZ could regulate downstream defense genes in an SA-independent signaling pathway, as exogenous applications of SA, MeJA, or ET did not activate its expression [115].

Research has shown that WRKY TFs can have a negative effect on viral infections. In *N. benthamiana*, the overexpression of NbWRKY40 resulted in a decrease in *tomato mosaic virus* (ToMV) accumulation, activation of the W-box promoter of ICS1 that facilitates SA synthesis, and upregulation of SA marker genes, PR1 and PR2 [116]. This is consistent with previous studies, suggesting that NbWRKY40-induced SA signaling may hamper the cell-to-cell movement of ToMV by facilitating callose deposition in plasmodesmata [117,118]. Similarly, the overexpression of WRKY30 in Arabidopsis resulted in higher resistance against CMV, while the wrky30 mutant line was more susceptible to CMV. The silencing of WRKY30-compromised brassinosteroid (BRs)-induced defense threatened the capacity of PSII photochemistry and led to a significantly higher ROS level. These findings suggest that WRKY30 may play a crucial role in BR-induced virus defense [119].

WRKY TFs have also been reported to exhibit antiviral activity in economically important crops. In citrus (*Poncirus trifoliate*) and pummelo (*Citrus grandis*) plants infected with *Citrus tristeza virus* (CTV), the expression of different WRKYs was altered [120]. The induction of PtrWRKY1 in CTV-inoculated citrus was greater than in pummelo, suggesting a role for PtrWRKY1 in resistance against CTV. In *C. annuum*, the expression CaWRKY-α was activated by several signal molecules, including SA, MeJA, and ethylene, and was hypothesized to regulate pathogen-induced defense mechanisms and participate in phytohormone crosstalk [31,121]. CaWRKYd, which is present in *C. annuum*, has been found to be stimulated by TMV and other phytohormones involved in plant defense. The suppression of CaWRKYd resulted in a decrease in HR cell death induced by TMV-P0 and an increase in virion accumulation. Additionally, CaWRKYd silencing led to a notable reduction in the expression of several genes related to HR and PR, such as CaBPR1, CaDEF1, CaAlaAT1, CaHIN1, and CaHIN2. The results indicate that CaWRKYd plays a critical role in resistance against TMV-P0 infection [122]. In onion (*Allium cepa*) infected with *onion yellow dwarf virus* (OYDV), AcWRKY32 was found to participate in transcriptomic reprogramming during dormant release and serve as a host factor adopted by OYDV for symptom development [123].

WRKY TFs from different plant species, including MtWRKY from *Medicago truncatula*, and GhWRKY15 and GhWRKY11 from *Gossypium hirsutum*, have been reported to provide resistance against TMV in *N. tabacum* [89,124]. Overexpression of these genes in transgenic tobacco plants activated the expression of several defense-related genes, such as PR, peroxidase (POD), and ascorbate peroxidase (APX), triggering systemic SAR to protect plants against TMV and CMV. The MtWRKY genes in tobacco exerted their regulatory roles in upregulating PR gene expression as well as lignin deposition against TMV [125]. In tobacco, NtWRKY1 was found to bind to one MAPK known as SA-induced protein kinase (SIKP) [126], which is activated following TMV infection [127]. The silencing of NtWRKY4-compromised antiviral defense resulted in a higher accumulation of TMV viral RNA in transgenic plants [128]. Similarly, AtWRKY6 was shown to have a dual function in viral infection support and inhibition [129], while AtWRKY8 plays a defensive role in response to crucifer-infecting TMV by regulating ET- and ABA-signaling crosstalk [130]. Furthermore, Chrysanthemum WRKY11 was found to be upregulated after infection by CMV, *tomato spotted wilt virus* (TSWV), and PVX [131]. The functional analysis of various WRKY TFs highlights their importance during pathogenic infections. 

### 3.4. bZIP Transcription Factor Family

**Figure 5:**
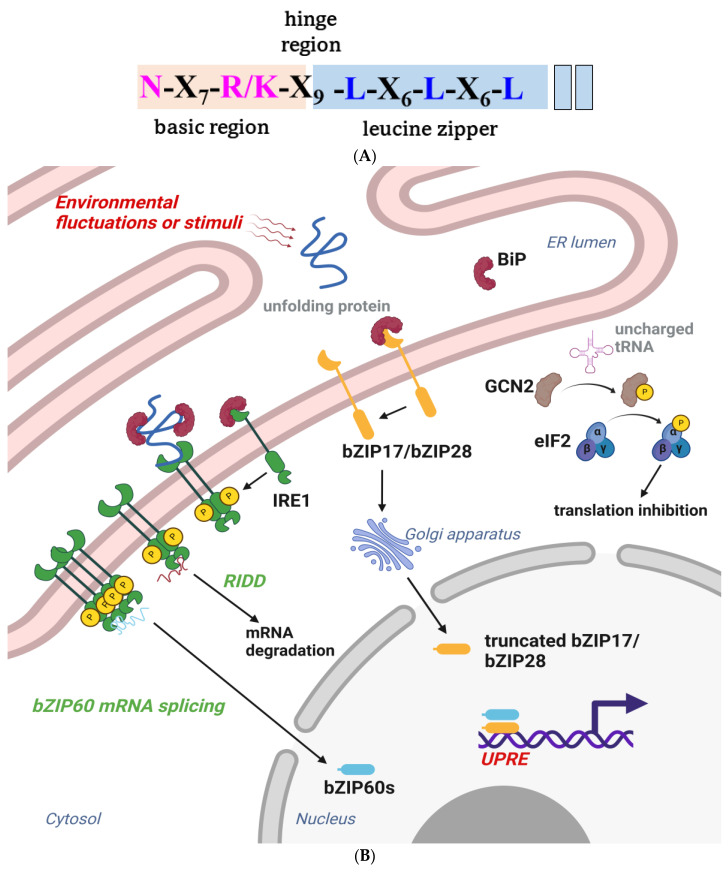
(**A**) The primary structure of the bZIP domain. The basic region is shaded in almond, the leucine zipper region is shaded with sky blue, and the highly conserved residues are highlighted with pink and blue. The leucines are sometimes replaced by isoleucine, valine, phenylalanine, or methionine. (**B**) A schematic of the participation of bZIP proteins in plant UPR branches. Plants have three major unfolded protein response (UPR) branches. The inositol-requiring enzyme type 1 (IRE1) branch pathway involves the activation of basic leucine zipper 60 (bZIP60) TF. A molecular chaperon resides in the ER lumen under homeostatic conditions; immunoglobulin-binding protein (BiP) binds to the luminal domain and inhibits IRE1 activities. ER stress leads to the dissociation of BiP from IRE1, thus activating IRE1 through oligomerization and trans-autophosphorylation. Activated IRE1 further splices bZIP60 (unspliced bZIP60, bZIP60u) mRNA via endonuclease activity and produces the spliced bZIP60 (bZIP60s) TF, which regulates several UPR target genes, including BiP, and brings about program cell death (PCD) [145,146]. IRE1 also mediates the bulk degradation of specific mRNA by Regulated IRE1-Dependent RNA Decay (RIDD). Several mRNAs or miRNAs have been identified as IRE1 substrates in the RIDD pathway, which eventually activates autophagy in response to ER stress. Although the mechanism of RIDD is largely unknown, studies have indicated that the dimerization of IRE1 may primarily manage the cleavage of target mRNA, rather than the oligomerization of IRE1, that prevails in IRE1-mediated splicing. Generally, IRE1 tunes pro-survival and pro-death pathways to determine cell fate during ER stress and has been described as a double-edged sword during ER stress [147,148]. The second branch of UPR is primarily constituted by bZIP17 and bZIP28. The dissociation of BiP from bZIP17/bZIP28 facilitates their translocation into the Golgi, where their transmembrane domains are spliced by Golgi-resident, site-specific proteases, S1P and S2P. The truncated transcription factors shuttle to the nucleus to upregulate the expression of molecular chaperones and other stress response genes. GCN2-mediated eIF2α phosphorylation in the third branch is primarily activated by amino acid starvation and involved in diverse stress conditions and antibacterial immunity [149,150,151,152]. The figure was created with BioRender.com.

Like other plant TFs, the basic leucine zipper (bZIP) belongs to a large family that modulates numerous physiological activities to help plants overcome biotic or abiotic stresses. The family was named based on the evolutionarily conserved bZIP domain, which is characterized by an N-terminal nuclear localization signal, an N-X7-R/K motif, and heptad repeats of leucines and other hydrophobic amino acids that form an amphipathic helix (Figure 5A). The contiguous helix can dimerize to form a zipper structure and bind to cognate DNA on the major groove [132,133]. Functional bZIPs can form either homodimers or heterodimers and bind to different promoters, which changes the plant transcriptome to adapt to different physiological conditions or protect it from pathogens. The bZIP proteins can only bind to specific cis-elements of stress-related genes, including the A-box, C-box, G-box, GLM, and PB-like sequences [134]. The ACGTCA consensus sequence in the promoter of PR1 was reported to be a recognition site for bZIP TF [135,136]. The TGA TFs are a bZIP group D subfamily, named after their ability, to bind to the TGACG motif. TGA2, TGA5, and TGA6 were previously associated with NPR1 in regulating the expression of PR1- and SA-induced pathogen resistance [137,138].

The role of bZIP in pathogen defense mechanisms has been increasingly studied. Octopine synthase (OCS)-like elements can be recognized by the ocs-element-binding factor (OBF) group of bZIP TF, and their association with EREBPs suggests their involvement in regulating ethylene-induced PR genes [139,140,141]. Lesions simulating disease resistance 1 (LSD1) has been reported to regulate ROS levels, which confer disease resistance and initiate programmed cell death (PCD). The interaction between AtbZIP10 and LSD1 results in the retention of AtbZIP10 in the cytosol and negatively affects the cell-death-related transcriptional activity of AtbZIP10 in initiating PCD [142,143,144].

Previous research revealed that AtNPR1 could interact with TGA2, TGA3, TGA5, and TGA6. Specifically, TGA2 and TGA3 were shown to recognize the SA-responsive element of the PR1 promoter, which includes the TGACG core sequence, a TGA-binding site. Mutations in npr1 negatively impact SA signaling as well as the interaction between NPR1 and TGAs, as reported in [153,154]. By contrast, overexpressing AtNPR1 in rice was found to enhance resistance against Xanthomonas oryzae pv. oryzae. Additionally, three rice bZIPs, that exhibit high similarity with AtTGA2, were found to interact with AtNPR1, using the yeast two-hybridization genetic method, potentially triggering SA signaling in rice, in a same manner to Arabidopsis [155]. Moreover, the D1 domain on the tobacco bZIP TF, BZI-1, has been found to mediate the interaction between BZI-1 and ankyrin-repeat protein (ANK1) [156]. This interaction may contribute to forming a protein complex and explain how ANK1 governs BZI-1’s function in auxin signaling and response to biotic stimuli.

Despite the limited number of investigations, some bZIPs have been identified as playing a role in virus resistance. For example, pepper-PMMV interaction 1 (PPI1), which encodes a protein with a DNA-binding domain that shares a high amino acid sequence similarity with the bZIP family, was differentially expressed in PMMV-infected *Capsicum chinense*. PPI1 transcripts were found to be upregulated during an incompatible interaction between pepper and pathogens, but not in response to abiotic compounds. The induction of PPI1 significantly inhibited viral or bacterial pathogen infection in capsicum leaves. The findings suggest that PPI1 responds specifically to biotic stress and participates in the plant defense-signaling pathway [157]. Additionally, Box II, a cis-element located in the promoter of *rice tungro bacilliform virus* (RTBV), a persistent pathogen that mainly infects rice fields in South Asia, has been identified. RF2a and RF2b from rice, which also share high sequence identity with the bZIP family, were shown to act as homo- and hetero-dimers in a distinct interaction with Box II. Their predominant localization in vascular tissue may be involved in the regulation of phloem-specific expression of the RTBV promoter. Further, silencing of RF2 in transgenic rice produces similar symptoms to those caused by RTBV. The results suggest that RTBV inhibits RF2a and RF2b expression in the phloem, while the presence of RF2b may disturb the normal physiological state and affect the symptom manifestation of rice tungro disease [158,159,160].

Positive-stranded RNA viruses establish a viral replication complex (VRC) from a membranous organelle, such as the endoplasmic reticulum (ER), to facilitate their replication and cell-to-cell movement. The sensors on the ER membrane can detect the increasing need for viral protein synthesis, triggering the unfolded protein response (UPR) to manage cytosol perturbations and activate cellular defenses [161,162]. Several bZIPs, such as bZIP17, bZIP28, and bZIP60, were found to be involved in UPR pathways (Figure 5B). Recent studies have suggested that functionally redundant homologues of IRE1a/IRE1b in Arabidopsis may have different roles in plant defense. Specifically, IRE1a-bZIP60, but not IRE1b, may contribute to resistance against *plantago asiatica mosaic virus* (PlAMV) accumulation in Arabidopsis, whereas both IRE1a/IRE1b and bZIP60 play critical roles in resistance against TuMV accumulation. Moreover, the triple gene block 3 (TGB3) from PlAMV, and 6K2 protein from TuMV and *Potato virus Y* (PVY), were recently reported to induce the expression of bZIP17, bZIP28, and bZIP60 [33,163,164], all of which use the same G-box sequence as a recognition site on the target promoter. However, the effects of bZIP17/bZIP60 and bZIP28/bZIP60 on each virus were quite distinct. Specifically, the defense against PIAMV was induced by the synergism of bZIP17/bZIP60, while the defense against TuMV was induced by the synergism of bZIP28/bZIP60. A deeper examination of the impact of UPR on virus replication or movement could provide additional insights into the antiviral properties mediated by these bZIPs. 

Homeodomain-leucine zipper protein 1 (HAT1) of Arabidopsis, belonging to the hdZIP family of TFs, has been shown to be critical in plant defense against viruses. Overexpression and knockout analyses of HAT1, during infection with CMV, demonstrated that the knockout mutants exhibited greater tolerance to infection than did the overexpressing lines. Additionally, antioxidant systems and gene transcription involved in defense were downregulated in HAT1 overexpression lines but upregulated in mutant lines. Therefore, these findings suggest that HAT1 serves as a negative regulator of plant defenses against CMV [165]. A recent study indicated that HAT1’s role in the immune response against CMV is dependent on SA but not JA. These findings suggest that bZIP TFs and SA signaling pathways can be used to generate virus-resistant plant genotypes [166].

### 3.5. AP2/ERF Transcription Factor Family

**Figure 6:**
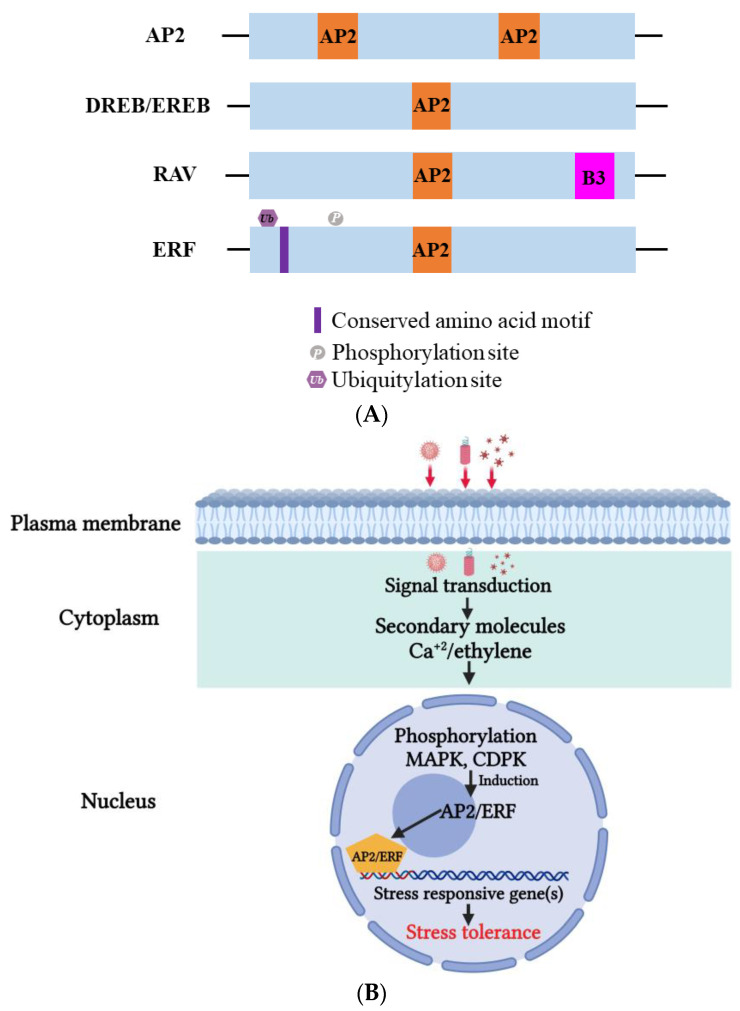
(**A**) Domain organization of Apetala2 (AP2), dehydration-responsive element-binding proteins (DREB), ethylene-responsive element-binding proteins (EREBs), ethylene-responsive element (ERF), and related to abscisic acid insensitive 3/viviparous 1 (RAV) proteins. Conserved amino acid motifs (AP2 and B3) that mark the different groups are indicated. (**B**) On viral infection, induction of AP2/ERF TFs takes place, which in turn regulates the expression of many defense-related genes such as pathogenesis-related (PR) genes. This induction also triggers small molecules such as calcium ions (Ca^+2^) and hormones such as ethylene to activate phosphorylation and subsequent defense response regulation. MAPK; mitogen-activated protein kinase, CDPK; calcium-dependent protein kinase.

Apetala2/ethylene-responsive factor (AP2/ERF) is a critical TF in plants that plays a versatile role in helping plants survive harsh environmental conditions such as heat, drought, and salinity [167,168]. Based on their highly conserved DNA-binding domains (DBDs), AP2/ERF proteins can be classified into four subfamilies: AP2, RAV (related to Abscisic acid insensitive3/Viviparous1), DREBs (dehydration-responsive element-binding proteins, subgroup A1–A6), and ERFs (ethylene responsive factor, subgroup B1–B6) (Figure 6A) [26,169,170]. Two amino acids, Asp14, and Ala9, participate in the cis-element binding process [26]. The AP2/ERF DBD consists of roughly 70 amino acids that create three beta-sheets and an almost parallel alpha-helix [170]. AP2/ERF can recognize the dehydration-responsive/c-repeat element (DRE/CRT), A/GCCGAC, or the ethylene-responsive element (ERE), AGCCGCC (GCG-box), on the promoter and regulate downstream gene expression [26,171,172]. The ERFs participate in biotic stress tolerance through the GCC box. The regulatory activities of AP2/ERF can also be influenced by the variant C-terminal domain. For example, a consensus EDLL motif, containing conserved Glu (E), Asp (D) and Leu (L), was discovered in the C-terminus of AtERF98, which functions as an activation domain that activates downstream genes. Conversely, an ERF-associated amphiphilic (EAR) motif or B3 repression domain (BRD) can suppress downstream gene expression [173,174,175]. The EAR motif also recruits specific co-repressors, such as TOPLESS (TPL), topless-related (TPR), and histone deacetylase 19 (HAD19), to regulate downstream gene expression [176,177]. Through the coordination of various elements, the AP2/ERF superfamily can help plants adapt to environmental changes and overcome stresses. The mechanism of AP2/ERF TFs is illustrated in Figure 6B.

Previous studies have demonstrated that AP2/ERF TFs play a role in pathogen defense, in response to biotic stress. In *N. tabacum*, ERF5 was found to be induced by wounds and infection from several pathogens, including TMV. The overexpression of ERF5 was reported to provide specific resistance against the replication and movement of TMV, through an N gene-independent mechanism [178]. A comprehensive analysis [32] revealed that several ERFs are differentially expressed in resistant and susceptible tomatoes during TYLCV infection. About 22 ERFs were modulated by TYLCV in tomato, and 5 ERF-B3 TFs were identified in tomato cultivars Hongbeibei, Zheza-301/Zhefen-702, and Jinpeng-1/Xianke-6 (highly resistant, resistant, and susceptible, respectively). The researchers also identified several ERFs with varying affinity for the GCC-box, which could assist in a variety of regulatory functions. These ERFs may regulate downstream proteins such as MAPK, which has been linked to the response to phytohormones and the regulation of WRRKY proteins. These findings provide a plausible pathway for explaining how tomato ERFs determine resistance against TYLCV [32]. In *N. benthamiana*, ERF5 (NbERF5) was characterized as a factor whose overexpression reduced viral accumulation. Protein–DNA interaction analysis revealed that NtERF5 interacted with the GCC box cis-elements, suggesting potential regulation of PR genes. Plants overexpressing ERF5 under the constitutive promoter, CaMV 35S, exhibited a higher level of resistance, a reduced HR response, and impaired systemic virus spread [178].

GLRaV-2 is a common virus associated with grapevine leafroll disease. The protein p24, which is an RNA-silencing suppressor (RSS) encoded by GLRaV-2, promotes the accumulation of GLRaV-2 by interacting with the B3 DNA-binding domain of grapevine related to abscisic acid insensitive3/viviparous1 (VvRAV1) transcription factor. Salicylic acid inducible VvRAV1 positively regulates the grapevine PR1 (VvPR1) gene by directly binding its promoter, suggesting that VvRAV1 may be involved in the regulation of host basal defense responses [179]. However, GLRaV-2 p24 has been shown to interact with and recruit VvRAV1 in the cytoplasm to bind 21-nt ds siRNA, thereby increasing its silencing suppression ability. At the same time, p24 enters the nucleus by interacting with VvRAV1 and reduces VvRAV1-mediated activation of VvPR1 transcription [180].

The important roles of AP2/ERF TFs in plants are widely recognized. With an increasing number of plant genomes being accessible in public databases, genome-based studies on AP2/ERF are becoming more prevalent. However, recent studies on AP2/ERF mainly focus on verifying gene function. The continued development of molecular biology techniques, including genome editing, will facilitate more convenient and in-depth research on AP2/ERF family factors.

## 4. Concluding Remarks

This review summarizes the current advances in the understanding of the roles of major plant TFs in the defense against biotic, particularly viral, stresses, and highlights the potential applications of these resistance/tolerance mechanisms. 

One of the most significant challenges facing humanity is guaranteeing food security. To meet the growing demand for food and sustain the expanding population, stress-tolerant crop varieties are critical. Plant biotic stress resistance not only enhances food security but also reduces the use of pesticides during crop production, leading to reduced production costs. TFs play a critical role at the transcriptional level, activating or inhibiting genes in response to various stresses. Among thousands of TFs identified in plants, the most important ones are distinct signal transduction pathways mediated by different TF families, such as NAC, WRKY, MYB, bZIP, AP2/ERF, etc. Crops have been exposed to various biotic and abiotic stress conditions over their evolutionary history, and they have developed physiological and molecular mechanisms, especially TFs, to cope with these challenges. Functional analysis of existing TFs has revealed that they can serve as targets for gene expression to control phytopathogenic viruses through using advanced disease management approaches. TFs can play a role in regulating various genes associated with defensive mechanisms, which have been studied in several model plants. Genetic and molecular techniques have greatly aided in the functional characterization of TFs. While detailed characterization tests have been carried out on Arabidopsis, tobacco, or rice, only a few members of the TFs have been functionally characterized in other species. In addition, there are still significant knowledge gaps between our understanding of these TFs and the actual applications. For example, the mechanisms for the crosstalks among the different pathways, regulated by various TFs under stress conditions, or the first elements of the surveillance system that sense the specific patterns or elicitors of the invading viruses in plants. As specific viruses seemed to only activate/repress the expression of specific TFs, there appear to be certain recognition systems in plants, like those in the immune systems of animals. These knowledge gaps require further studies to lead the way to practical applications. Nonetheless, the current investigations have provided excellent insights into viral stress signaling and response networks, including TFs.

Various TF-regulated signal transduction pathways have been examined with the goal of alleviating viral stresses through control and modification. The use of TFs enables the plant to modify its surroundings, protecting it from a range of insects and pathogens. Although viral infections are challenging, TF regulation can be an important tool in protecting plants from viral stress. Tight control and fine-tuning of TFs during plant stress responses, involving interactions between TF proteins, downstream targets, and upstream regulators, contribute to the formation of complex signaling webs that play a role in stress modulation. For decades, breeders have attempted to improve the quality of crops through different approaches. The current development in the modulation of TFs may present novel tools and strategies for dealing with viral stresses and biotic stresses in general. Over time, as more and more elements are being discovered, the applications of TF expression manipulation in crop improvement may finally become a reality.
